# Effect of High Fiber Cereal Intake on Satiety and Gastrointestinal Symptoms during Ramadan

**DOI:** 10.3390/nu11040939

**Published:** 2019-04-25

**Authors:** Amjad H. Jarrar, Jeannette M. Beasley, Eric O. Ohuma, Leila Cheikh Ismail, Dina A. Qeshta, Maysm N. Mohamad, Ayesha S. Al Dhaheri

**Affiliations:** 1 Nutrition and Health Department, College of Food and Agriculture, United Arab Emirates University, Al Ain 15551, UAE; amjadj@uaeu.ac.ae (A.H.J.); d.qeshta@outlook.com (D.A.Q.); maysmnezar88@uaeu.ac.ae (M.N.M.); 2 Department of Medicine, New York University, New York, NY 10016, USA; Jeannette.beasley@nyulangone.org; 3 Centre for Tropical Medicine and Global Health, Nuffield Department of Medicine University of Oxford, Old Road Campus, Oxford OX3 7BN, UK; eric.ohuma@ndm.ox.ac.uk; 4 Clinical Nutrition and Dietetics Department, College of Health Sciences, University of Sharjah, Sharjah 27272, UAE; lcheikhismail@sharjah.ac.ae

**Keywords:** dietary fiber, Ramadan, cereal, high fiber

## Abstract

(1) Background: Fasting during Ramadan involves large changes in daily eating patterns which strongly impacts the daily biorhythm and challenges the regular function of the digestive tract. The aim of this study was to assess satiety, bowel habits, body composition, blood glycaemia, and blood lipidemia after the consumption of high fiber cereal at dawn (Sohor) during the month of Ramadan; (2) Methods: A two-arm randomized, controlled, single-blinded, parallel-design study was conducted in Ramadan month. Participants were randomized to consume either 90 g of high fiber cereal (11 g fiber/90 g) at Sohor for 20 consecutive days (intervention group, *n* = 45) or to maintain their habitual diet intake (control group; *n* = 36); (3) Results: The intervention group reported higher satiety rating scores, improved bowel habits and reduced bloating frequency after the 20-day intervention. Significantly higher intake of carbohydrates and dietary fiber were observed in the intervention group. Total cholesterol and low density lipoprotein (LDL) cholesterol were significantly lower among the intervention group compared to the control group (*p*-value = 0.043, and *p*-value = 0.033, respectively) at the end of the intervention. No significant differences in body weight, body fat percentage, waist circumference, body mass index, blood glucose, high density lipoprotein (HDL) cholesterol, and triglycerides were observed between the two groups; (4) Conclusions: Consuming high fiber cereal had a positive effect on health and well-being during the month of Ramadan with better satiety, improved bowel functions, and improved blood lipids.

## 1. Introduction

There are several definitions for dietary fibers (DF), described as non-starch polysaccharides (NSP), from around the world including those from CODEX, the U.S. Institute of Medicine (IOM), Health Canada, European Food Safety Authority (EFSA), Food Standards Australia and New Zealand (FSANZ), and the American Association of Cereal Chemists International (AACCI) [1,2,3].

All DF definitions distinguish non-digested fibrous materials naturally occurring in food as part of the fiber complex. Many definitions also recognize these non-digested carbohydrate (CHO) materials, when they are extracted from edible material, synthesized, or modified and added back to the food [1].

Despite the different definitions of DF, most definitions require at least one physiological benefit for adding fibers to food, such as improved intestinal transit time and increased stool bulk; fermentation by colonic microflora; reduction in blood total and/or low density lipoprotein (LDL) cholesterol levels; and reduction in post-prandial blood glucose and/or insulin levels [1,3,4].

Several studies have shown that DF induces greater satiety than digestible polysaccharides and simple sugars [5]. Satiety can be achieved via several factors, including the intrinsic physical properties of DF (bulking, gel formation, and viscosity change of gastric contents) [6], and modulation of gastric motor function and blunting of postprandial glucose and insulin responses. DF may also prolong meal duration and result in increased mastication with possible cephalic and peripheral influences on satiety [7]. DF-containing meals have a lower energy density [5] and may affect palatability of food, possibly reducing energy intake [8].

A review of the role of DF in energy regulation reports that different types of DF appear to either affect early signals of satiation or enhance satiety [9].

Many studies have reported a role for breakfast cereal consumption in a balanced diet [10,11,12]. Dietary guidelines note that the high nutrient density of breakfast cereals (especially those that are whole grain or high in cereal fiber) makes them an important source of key nutrients [13]. Regular breakfast cereal consumption is associated with a lower body mass index (BMI), lower risk of diabetes and cardiovascular disease, and a greater feeling of well-being [14]. However, there is lack of research on the effect of high fiber breakfast cereal consumption and gastrointestinal symptoms during Ramadan.

Ramadan is the ninth month of the Islamic lunar calendar, and a month when Muslims refrain from drinking, eating, and smoking from dawn (Sohor) until sunset (Iftar). During the month of Ramadan Muslims consume two meals in a day, causing dramatic changes to their nutritional habits. These changes lead to differences in body weight [15], energy balance [16], and some biochemical parameters when compared to those measured before Ramadan. Food consumed during this month is normally high in protein and fat. Accordingly, dyspeptic symptoms are frequently encountered during Ramadan, with indigestion, bloating, and heartburn (gastroesophageal reflux) being more common. There has been only one study to date on the prevalence of gastrointestinal symptoms during Ramadan [17]. Belching, bloating, and a fullness sensation were reported to be the most common symptoms, with an overall prevalence of 19.9%, while the least prevalent symptom was diarrhea with a prevalence of 0.6%. Other gastrointestinal symptoms and their prevalence were as follows: mouth dryness and bitter taste in the mouth (18.7%), epigastric pain and discomfort (11.7%), early starvation (10.5%), early satiety (9.9%), loss of appetite (8.8%), heartburn (5.3%), abdominal pain, flank pain and periumbilical pain (5.3%), constipation (5.3%), and nausea and vomiting (4%). Most of the upper gastrointestinal symptoms were found to correlate with dietary components such as carbohydrate, fat, and protein intake [17]. The current study aimed to assess the effect of high fiber cereal consumption on satiety, nutrient and energy intake, lipid profile, and glycemic responses and gastrointestinal symptoms during Ramadan.

## 2. Materials and Methods

### 2.1. Study Design

This research study was a randomized, controlled, single-blinded, parallel-design study. High fiber cereal with 11 g fiber per ~90 g of product was given to participants, and the effects on blood glycemia, blood lipidemia, satiety, bowel function, bloating, and body composition were measured. The study began on the 3rd day of Ramadan (June) 2016 and the intervention duration was 20 days.

### 2.2. Research Participants

A total of 81 participants were recruited through face-to-face interviews, posters at the United Arab Emirates University (UAEU), and via email. Participants were provided with both verbal and written information (participant information sheet) about the project’s aims, data to be collected, and study duration. A screening questionnaire was administered to participants to collect data on their medical history, medication use, and current health status. All participants signed an informed consent form to voluntarily participate in the study. This study was conducted according to the guidelines in the Declaration of Helsinki and all procedures involving human subjects were approved by the United Arab Emirates University (UAEU) Scientific Research Ethics Committee (Reference Number ERH_2016_4372).

### 2.3. Inclusion and Exclusion Criteria

The study excluded any participants who self-reported having chronic diseases, such as heart disease, hypertension, renal failure, or liver disease, participants treated with diuretics or laxatives, and pregnant or lactating women. The study included any student or staff member of the UAEU aged between 18 and 65 years with a BMI of 18.5–30 kg/m^2^, who fasted during the month of Ramadan in 2016 and who did not object to consuming high fiber cereal at the Sohor meal daily.

### 2.4. Research Procedures

A 20-day randomized intervention was conducted by providing the intervention group (*n* = 45) with high fiber cereal (90 g of product containing 11 g of dietary fiber, 60 g of carbohydrates, 10.5 g of protein, and 2.8 g of fat) to consume daily at the Sohor meal and comparing the results with those of a control group maintaining their habitual diets (*n* = 36; no consumption of high fiber cereal). The aim of this study was to assess the effect of high fiber cereal on increasing satiety and as a result facilitating weight reduction. The study was designed to detect a 1.5-kg difference in weight loss between the two groups at the end of the 20 days trial. Assuming a common SD of 2.2 kg, we needed to enroll 68 participants for the study to have a power of 80% (beta = 0.20) for a 2-sided (1:1 ratio) test with a type I error rate of 0.05 [18]. The RAND function from Microsoft Excel was used to randomize participants into the two groups. The intervention began on the 3rd day of Ramadan, with baseline (intervention day 0) and endpoint (intervention day 20) measurements collected.

### 2.5. Anthropometric Measurements

Body weight was recorded to the nearest 0.01 kg while the subject was wearing minimal clothing and no shoes. Body composition was analyzed using an InBody720 body composition analyzer (Biospace Co., Seoul, Korea) for the measurement of body fat (%), fat mass (kg), and fat free mass (kg). Waist circumference was measured using a measuring tape. Anthropometric measurements were taken three times and an average value was recorded. All measurements were collected at baseline and endpoint.

### 2.6. Diet and Physical Activity Assessment

Participants were asked to maintain their normal Ramadan lifestyle during the study period and were asked to record their dietary intake using 3-day food records at baseline and endpoint. Each 3-day food record included two weekdays and one weekend day. Food photographs with different portion sizes were provided and used by the participants to help estimate the amount of food consumed. The Nutrition Analysis Software program (The Food Processor, version 10.4, ESHA Research, Salem, OR, USA) and the Kuwaiti Food Composition database were used to estimate the energy and nutrient content of consumed foods.

### 2.7. Gastrointestinal Symptom and Satiety Questionnaires

Participants were given a gastrointestinal symptom questionnaire to assess the frequency and intensity of constipation, bloating, diarrhea, and heartburn at baseline and endpoint of Ramadan. Furthermore, a satiety rating scale was used at endpoint to assess satiety immediately at wakeup, before the Sohor meal, 15 min after ingestion of the Sohor meal, at 12:00 pm, 2 h prior the Iftar meal, and at the Iftar meal. The subjective feeling of satiety was measured using an equilateral three-point rating scale (adapted from Holt et al., 1995) [19]. The scale was anchored at −3 (‘hungry’; having a strong desire for food) with a midpoint at 0 (‘neutral’; neither hungry nor full) through to +3 (‘satisfied’; feeling of fullness and suppression of hunger).

### 2.8. Biochemical Parameters

Fasting blood glucose, triglycerides, high density lipoprotein (HDL) cholesterol, low density lipoprotein (LDL) cholesterol, and total cholesterol were analyzed using the Cobas C111 automated biochemical analyzer (Roche Diagnostics, Indianapolis, IN, USA). Venous blood samples (5 mL) were collected after 12 h of fasting by a trained and registered phlebotomist in the Nutrition and Health Department. Blood collection was done using a vacuum system (vacuette 0.64 Å ~ 19mm, Greiner Bio-One, Kremsmünster, Austria), into a serum separator tube with a clot activator (Vacutest Kima srl, Arzergrande, Italy). Blood samples were centrifuged (2500 rpm, 15 min) and the serum was appropriately separated, identified, and stored at −80 °C until the time of analysis. Biochemical measurements were collected at baseline and endpoint for both control and intervention groups.

### 2.9. Statistical Analysis

Descriptive statistics and frequency checks summarized variables and checked for missing values. A Chi-square test and Fishers exact test (when the total per cell was less than 5) were used where appropriate to assess associations between any two independent parameters. Further, a Chi-square test was used to compare the ‘satisfied’ groups between the control and intervention groups. Student’s t-test was used to compare mean differences between any two groups. Statistical significance was assessed at the 5% level (*p*-value < 0.05). All analyses were performed using STATA version 11.2 (StataCorp, College Station, TX, USA).

## 3. Results

### 3.1. Characteristics of the Study Population

Participants (*n* = 81) ranged in age from 18 to 47 years, and the mean age was 22.1 ± 4.4 years. Almost all (94%) participants were females (Table 1). By the end of the study period, the control and the intervention groups showed a reduction in weight (*p*-value = 0.002 and 0.003, respectively) and BMI (*p*-value = 0.002 and 0.004, respectively). However, the overall change in weight and BMI between the control and the intervention group was not statistically significant (*p*-value = 0.667 and 0.890, respectively). Moreover, there was no significant change in the body fat percentage and waist circumference between the two groups over the course of the study.

### 3.2. Gastrointestinal Symptoms

Table 2 presents gastrointestinal symptoms experienced by control and intervention groups. At the endpoint of the current study, 49% of the intervention group reported an increase in bowel habits compared with 25% of the control group (*p*-value = 0.029; Table 2). The intervention group reported less constipation than the control group, with 4% of the intervention group reporting constipation in the last week of Ramadan compared with 8% of the control group, however, the difference is not significant. The intervention group reported significantly less bloating frequency compared to the control group (*p*-value < 0.001).

### 3.3. Satiety

**Figure 1:**
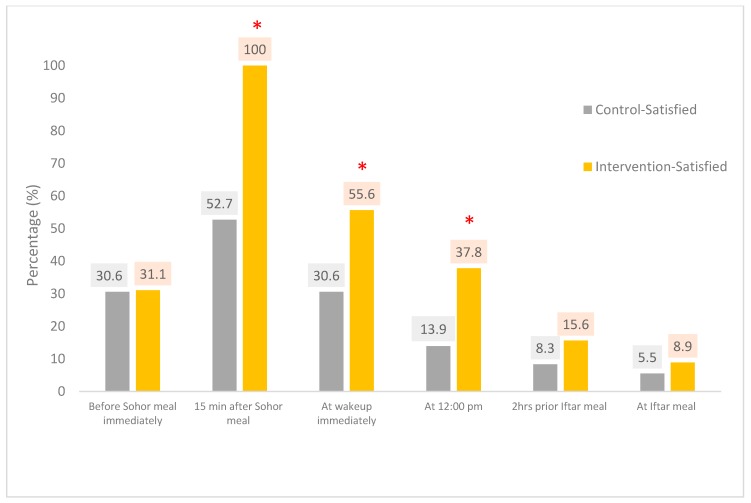
Subjective satiety rating for ‘satisfied’ for the control and intervention groups at the endpoint of Ramadan (* *p*-value < 0.05).

The satiety rating scale was used to measure the degree of satiety at different times of the day at the study endpoint for intervention and control groups. Fifteen minutes after ingestion of the Sohor meal, 100% of the intervention group were satisfied compared with 52.7% of the control group (*p*-value < 0.001; Table 3 and Figure 1). Two hours prior to the Iftar meal, 15.6% of the intervention group were satisfied compared with 8.3% of the control group (*p*-value = 0.865).

### 3.4. Diet Records

Three-day food records (two weekdays, one weekend day) were taken at baseline and endpoint from control and intervention groups. Table 4 shows the energy and macronutrient intake of the control and intervention groups at baseline and endpoint during Ramadan. Data indicated no significant difference in the dietary intake between the baseline and endpoint among participants in the control group and in the intervention group. However, results show significantly higher intake of carbohydrates and dietary fiber in the intervention group compared to the control group; which might be due to supplementing the intervention group participants with a high fiber cereal (90 g of product containing 11 g of dietary fiber and 60 g of carbohydrates) to consume daily at the Sohor meal, while the control group participants were asked to maintain their usual dietary intake.

### 3.5. Biochemical Measurements

Blood glucose, total cholesterol, HDL cholesterol, LDL cholesterol, and triglycerides were measured for control and intervention groups at baseline and endpoint (Table 5). Total cholesterol and LDL cholesterol significantly increased between the baseline and the endpoint within the control group (*p*-value = 0.012 and *p*-value = 0.006, respectively). However, no significant changes in biochemical measurements were seen within the intervention group.

Overall, the intervention group showed a significantly lower increase in total cholesterol and LDL cholesterol compared to the control group (*p*-value = 0.043 and *p*-value = 0.033, respectively).

## 4. Discussion

Scientific evidence from observational studies consistently proves that increasing the intake of fruits, vegetables, whole grains, and legumes is associated with greater weight loss over time [4,20,21,22]. However, several randomized controlled trials based on either an increase of fruit/vegetable consumption or high wholegrain food intake, showed no improvement in body weight or body fat [23,24,25,26]. On the other hand, many studies showed an association between increased dietary fiber intake from whole foods or supplemental fiber and body weight loss [27,28,29]. Several mechanisms were proposed to explain how increased fiber intake could lead to less energy intake: (1) calories and nutrients are replaced by fiber; (2) fiber needs more chewing, which reduces intake by stimulating the secretion of saliva and gastric juice, which in turn causes an expansion of the stomach and promotes satiety; and (3) fiber reduces absorption in the small intestine [30].

The current study demonstrated no statistically significant effects of high fiber cereal intake during Ramadan on body weight, percentage body fat, waist circumference, and BMI. Similarly, several intervention studies that studied the effect of adding cereal at breakfast did not show any significant improvement of weight measures [31,32,33,34]. Moreover, similar studies conducted during the month of Ramadan have also reported no significant changes in body weight and body fat [35,36]. This suggests either that the length, intensity, etc., of the intervention was not long enough to observe meaningful effects on body weight and body fat or that this type of dietary changes alone is not sufficient to promote these outcomes [37].

The current study suggests a positive effect of high fiber cereal consumption on reducing bloating and improving bowel movement frequency. Likewise, a recent systematic review of interventions studying the effect of high fiber cereal on bowel function indicated that wheat fiber promotes healthy bowel function as it improves total stool weight, dry stool weight, intestinal transit time, and stool frequency [38]. In addition, other studies conducted on Ramadan fasting individuals reported constipation as the most frequent gastrointestinal symptom experienced by the end of Ramadan [39,40]. Therefore, recommending high fiber cereal to reduce symptoms of constipation and improve bowel function for fasting individuals during Ramadan should be taken into consideration.

Participants from the intervention group (consumed high fiber cereal right before fasting) in the current study reported higher satiety rating scores throughout the time of fasting compared to the control group. A review conducted by Pereira and Ludwig revised 27 experimental studies that investigated the effect of dietary fiber on satiety [5]. Seventeen studies reported a positive effect of dietary fiber consumption on satiety and energy intake. Seven studies showed mixed effects, while only three articles reported no link of dietary fiber intake on satiety [5]. Different mechanisms were proposed to explain the effect of dietary fiber on satiety. Saris (2003), proposed that high fiber foods are usually less energy dense and larger in volume than low fiber foods, thus they take a longer time to digest and take up more space in the stomach which brings a feeling of fullness sooner [41].

With respect to food intakes during the current study period, the results show significantly higher intake of carbohydrate and fiber among the intervention group compared to the control group. This is because the intervention group participants were requested to consume 90 g of the high fiber cereal (containing 11 g of dietary fiber and 60 g of carbohydrates) at the Sohor meal daily. Therefore, the intervention group consumed 102% of the recommended dietary allowance (RDA) for dietary fiber intake compared to 74% of the RDA for the control group. Similarly, an Iranian study reported an average dietary fiber intake of 15 to 18 g/day among participants during Ramadan, which is comparable to dietary fiber intake of the control group in the current study indicating a habit of low dietary fiber consumption among fasting individuals during the month of Ramadan [42].

Many studies have strongly supported the hypothesis that whole grains, especially cereal fiber, reduce mortality, cardiovascular disease (CVD), and type 2 diabetes [4,20,43,44]. The Zutphen Cohort Study (40 year study) concluded that a 10 g increase in fiber intake reduced all-cause mortality by 9% and CVD mortality by 17% [45]. The Nurses’ Health Study (8 year study) showed a strong association between high fiber consumption and reducing the risk of diabetes [46]. Likewise, the current study showed a significant decrease of total cholesterol in the intervention group compared to the control group and a significantly lower increase in the LDL cholesterol compared to that of the control group. Over the course of this study, the plasma glucose, HDL cholesterol, and triglycerides did not change. The lack of response might be due to the short length of the intervention (only 20 days) or it could be related to the choice of healthy participants, rather than hypercholesterolaemic or diabetic subjects. Another limitation of the study is that the majority of our sample were females, with the fact that participants were voluntarily enrolled in the study, which could have caused selection bias.

## 5. Conclusions

An addition of 11 g of DF from high fiber cereal to the diet of the intervention group was enough for these participants to achieve the RDA of DF, whereas the control group did not meet this target. High fiber cereal consumption resulted in a positive effect on the reduction of bloating frequency and improvements in bowel movement. High fiber cereal consumption was linked to greater satiety rating scores among the intervention group compared to the control group during the time of fasting. There was no significant effect of the addition of DF to the diet on body weight, BMI, waist circumference, or percentage body fat during the 20-day study period. Consuming high fiber cereal showed a positive effect in maintaining total cholesterol and LDL cholesterol levels among the intervention group.

## Figures and Tables

**Table 1:** Physical characteristics of the study population (*n* = 81).

	Control (*n* = 36, 31 Females)			Intervention (*n* = 45, 45 Females)			Overall *p*-Value
Age (Year)	21.5 ± 1.9			22.6 ± 5.7			0.289
Height (cm)	161.1 ± 8.5			161.3 ± 5.6			0.888
	**Baseline** **M ± SD**	**Endpoint** **M ± SD**	***p*-Value**	**Baseline** **M ± SD**	**Endpoint** **M ± SD**	***p*-Value**	
Weight (kg)	60.5 ± 11.5	59.9 ± 11.2	0.002	62.5 ±11.1	62.0 ± 11.1	0.003	0.667
BMI (kg/cm²)	23.2 ± 3.6	23.0 ± 3.6	0.002	24.0 ± 3.9	23.8 ± 3.9	0.004	0.890
WC (cm)	73.8 ± 9.0	74.9 ± 8.4	0.053	75.1 ± 8.0	75.6 ± 8.7	0.221	0.391
% Body fat	31.0 ±8.9	30.4 ± 9.0	0.059	34.0 ± 7.1	34.1 ± 7.3	0.808	0.141

BMI: body mass index; WC: waist circumference; M: mean; SD: standard deviation; % Body fat: body fat percentage.

**Table 2:** Gastrointestinal symptoms experienced at the endpoint of Ramadan.

Gastrointestinal Symptoms	Response/Frequency	Control (*n* = 36) Count (%)	Intervention (*n* = 45) Count (%)	*p*-Value
Bowel habits increase	Yes	9 (25)	22 (49)	0.029
	No	27 (75)	23 (51)	
Constipation	Yes	3 (8)	2 (4)	0.417
Bowel movement frequency	≤1/week	6 (16.6)	4 (9)	0.294
	2/week	8 (22.2)	12 (27)	
	≥3/week	22 (61.1)	29 (64)	
Vomiting frequency	None	23 (64)	34 (75.5)	0.467
	1–2/week	13 (36)	11 (24.5)	
Bloating frequency	≤1/week	18 (50)	28 (62.2)	<0.001
	2/week	13 (36)	12 (26.6)	
	≥3/week	5 (14)	5 (11.1)	
Heartburn frequency	≤1/week	23 (64)	29 (64.4)	0.418
	2/week	9 (25)	12 (26.6)	
	≥3/week	4 (11)	4 (9)	

**Table 3:** Satiety scale at the endpoint of Ramadan for control and intervention groups.

Time	Control (*n* = 36) Count (%)			Intervention (*n* = 45) Count (%)		
	Neutral	Hungry	Satisfied	Neutral	Hungry	Satisfied
Before Sohor meal immediately	6 (16.6)	19 (52.8)	11 (30.6)	9 (20.0)	22 (48.9)	14 (31.1)
15 min after Sohor meal	16 (44.4)	1 (3)	19 (52.7)	0 (0)	0 (0)	45 (100)
At wakeup immediately	19 (52.7)	6 (16.7)	11 (30.6)	15 (33.3)	5 (11.1)	25 (55.6)
At 12:00 pm	12 (33.3)	19 (52.8)	5 (13.9)	18 (40.0)	10 (22.2)	17 (37.8)
2 h prior Iftar meal	6 (16.7)	27 (75.0)	3 (8.3)	9 (20)	29 (64.4)	7 (15.6)
At Iftar meal	7 (19.4)	27 (75.0)	2 (5.5)	7 (15.5)	34 (75.6)	4 (8.9)

**Table 4:** Macronutrient intake of the control and intervention groups at baseline and endpoint during Ramadan.

	Control (*n* = 36)			Intervention (*n* = 45)			Overall *p*-Value
	Baseline M ± SD	Endpoint M ± SD	*p*-Value	Baseline M ± SD	Endpoint M ± SD	*p*-Value	
Energy (kcal)	1913 ± 154	1876 ± 123	0.480	2021 ± 108	1939 ± 71	0.480	0.957
Carbohydrate (g) (% of total kcal)	244 ± 30 (51%)	220 ± 16 (47%)	0.343	266 ± 8 (53%)	264 ±11 (55%)	0.602	0.048
Protein (g) (% of total kcal)	64.4 ± 4.9 (14%)	66.4 ± 4.6 (14%)	0.935	66.1 ± 4.8 (13%)	60.9 ± 2.6 (13%)	0.348	0.931
Fat (g) (% of total kcal)	68.9 ± 5.5 (33%)	74.8 ± 6.3 (36%)	0.431	69.2 ± 7.9 (31%)	65.4 ± 3.9 (30%)	0.667	0.784
Dietary fiber (g) (% of RDA)	18.5 ± 1.8 (74%)	18.6 ± 1.3 (74%)	0.901	17.8 ± 1.7 (71%)	25.6 ± 1. (102%)	<0.001	<0.001

% of total Kcal: percent of total calories; % of RDA: percent of recommended daily allowance; M: mean; SD: standard deviation.

**Table 5:** Biochemical measurements of the study population.

	Control (*n* = 36)			Intervention (*n* = 45)			Overall *p*-Value
	Baseline M ± SD	Endpoint M ± SD	*p*-Value	Baseline M ± SD	Endpoint M ± SD	*p*-Value	
Glucose (mg)	85.7 ± 11.0	89.9 ± 12.2	0.092	83.7 ± 13.5	83.6 ± 15.2	0.991	0.271
Total cholesterol (mg)	149.5 ± 32.3	167.7 ± 33.6	0.012	140.7 ± 34.4	138.8 ± 42.8	0.778	0.043
HDL cholesterol (mg)	53.3 ± 14.8	53.5 ± 13.7	0.921	50.6 ± 14.3	51.1 ± 15.3	0.815	0.922
LDL cholesterol (mg)	81.6 ± 23.8	96.3 ± 26.3	0.006	77.6 ± 29.0	78.7 ± 28.6	0.786	0.033
Triglycerides (mg)	65.8 ± 18.9	77.1 ± 32.7	0.083	64.2 ± 19.8	71.5 ± 31.6	0.180	0.631

HDL: high density lipoprotein; LDL: low density lipoprotein; M: mean; SD: standard deviation.
